# IN TIME: IMPORTANCE OF OMEGA 3 IN CHILDREN’S NUTRITION

**DOI:** 10.1590/1984-0462/;2017;35;1;00018

**Published:** 2017

**Authors:** Francisca Echeverría González, Rodrigo Valenzuela Báez

**Affiliations:** a Departamento de Nutrição, Faculdade de Medicina, Universidad de Chile, Santiago, Chile.

Long-chain polyunsaturated fatty acids (LCPUFA), especially those of the n-3 family, such as eicosapentaenoic acid (C20:5 n-3, EPA) and docosahexaenoic acid (C22:6 n-3, DHA), have relevant biochemistry and physiological functions in human metabolism and health. In this regard, DHA is a fundamental nutrient for children’s growth and development. DHA has a key role in the formation and function of the central nervous system and the retina of humans.

This fatty acid is almost exclusively present, in significant amount, in diverse seafood (fish, shellfish, micro- and macroalgae). In fact, the intake of aquatic foods during the mid-Upper Paleolithic marked a significant turning point in human evolution.^1^ Additionally to the evolutionary importance of DHA for our specie, research established that n-3 LCPUFAs are critical during pregnancy and the early stage of childhood when DHA particularly plays a crucial role in brain and eye development and function.^2^ Deficiencies and imbalances of LCPUFAs (n-6 and n-3) are positively correlated with impairments in cognitive and behavioral performance of the child.^3^ Accordingly, the nutritional status of DHA during pregnancy and lactation periods represents a critical step for the brain and visual development of the child. It has been demonstrated that high plasma levels of DHA in the mother, and particularly in breast milk, are directly correlated with better growth and development of the brain and visual system in children.^4^ Formulas that supply a minimum of 0.35% DHA favor a better brain development, evaluated as Mental Development Index, which supports that the early dietary supply of DHA significantly improves mental performance.^5^ Children of 9 and 12 months of age supplemented with fish oil (natural source of EPA and DHA) have shown an effect of n-3 LCPUFA on their attention scores in a free-play test. On the other hand, the supplementation resulted in a decrease of systolic blood pressure, so the consumption of n-3 LCPUFA in the second half of infancy could have cognitive, as well as cardiovascular, beneficial effects.^6^ Healthy 4-year-old children, supplemented with 400 mg/d of DHA for 4 months, were evaluated in a randomized, placebo-controlled, double-blind study. The regression analysis showed a significant positive association between the blood level of DHA and the test of listening comprehension and vocabulary acquisition.^7^ A randomized controlled trial to determine the effects of an EPA-rich oil and a DHA-rich oil versus a safflower oil in children of 7 to 12 years old with attention-deficit/hyperactivity disorder (ADHD) showed that an increase in the erythrocyte DHA content was associated with improved lecture capacity and oppositional behavior. Moreover, increased EPA and total n-3 LCPUFA levels were associated with decreased anxiety/shyness. Accordingly, the supplementation with n-3 LCPUFA was associated with improvements in literacy and behavior of children with ADHD.^8^ Maturation of visual acuity was evaluated in children between 6 and 12 months of age. Breast-fed infants were randomly assigned to receive baby food containing egg yolk enriched with DHA or control baby food. Those infants fed with the n-3 LCPUFA enriched baby food have shown more mature visual-evoked potential acuity than the control group.^9^ This suggests that DHA intake for visual maturation is not only important during the perinatal period, but is also necessary until one year of life.^9^ On the other hand, infants fed with formulas supplemented with DHA and arachidonic acid (C20:4 n-6, ARA) have immune cell distribution and cytokine profiles similar to those of human milk fed infants. Foiles et al.^10^ followed a 91 healthy children cohort during a 6-year period and concluded that supplementation of infant formula with DHA and ARA in the first year of life delays allergy and has a protective effect against allergies in early childhood. Furthermore, premature infants could beneficiate as well from DHA supplementation. A double-blind, randomized, controlled trial determined feasibility, tolerability and efficacy of daily enteral supplementation (50 mg/d) in addition to standard nutrition for preterm infants (24-34 weeks gestational age). Those preterm infants who received supplementation had a progressive increase in circulating DHA, which suggests that daily enteral DHA supplementation is feasible and reduces deficiency in premature infants.^11^

In conclusion, n-3 LCPUFA have a key role in children’s growth and development, with special implications in:

- the central nervous system, showing improvements in different parameters of cognitive function;
- visual development, resulting in a better visual acuity;
- cardiovascular health, improving blood pressure; and
- the immune system, protecting the child against allergies in early childhood.

Therefore, an adequate intake of these fatty acids should be ensured from pregnancy, lactation and during childhood, and supplementation with n-3 LCPUFA should be considered when dietary intake is not sufficient or when one of the pathologies described above is present.
